# Adaptive radiotherapy incident-informed team training: a development and feasibility study

**DOI:** 10.1016/j.tipsro.2026.100407

**Published:** 2026-04-22

**Authors:** Farnoush Forghani, Sophie Lammers, Robbie Beckert, Dean Hobbis, Amanda Jackson, Beatriz Guevara, Thomas Mazur, Geoffrey D. Hugo, Eric Laugeman, Alex Price

**Affiliations:** a Department of Radiation Oncology, WashU Medicine, St. Louis, MO, USA; b University of Pennsylvania, College of Arts and Sciences, Philadelphia, PA, USA; c Department of Radiation Oncology, Case Western Reserve University School of Medicine, Cleveland, OH, USA

## Abstract

• Novel multi-institutional, incident-informed ART team training for patient safety.
• Real ART incidents recreated using retrospective patient treatment data.
• Incidents used for educational materials to teach error recognition and mitigation to ART team.
• First study on incident-based training and credentialing for online ART.

## Introduction

Adaptive radiotherapy relies on a highly complex workflow to generate daily treatment plans that account for inter-fraction anatomical changes [1], [2]. Because of this complexity, ART workflows are often developed using Failure Mode and Effects Analysis (FMEA), a risk-based framework designed to enhance quality and safety [3], [4], [5], [6]. Nevertheless, incidents can still occur, highlighting the need for a robust quality assurance management system to mitigate their impact on patients [7], [8], [9]. The clinical importance of minimizing radiotherapy deviations has been demonstrated in several multicenter trials. For example, analyses from the RTOG 9704 trial in pancreatic cancer patients linked failure to adhere to protocol-specified radiation therapy guidelines with decreased survival [10]. Similarly, the EORTC STRASS trial failed to demonstrate a survival advantage for neoadjuvant radiotherapy compared with surgery alone in retroperitoneal sarcoma. A subsequent radiotherapy quality assurance review identified target coverage deviations in approximately 25% of cases, emphasizing the clinical importance of treatment protocol adherence [11]. These findings highlight the clinical impact of treatment deviations and underscore the need for proactive quality assurance and incident-informed learning, particularly in complex workflows such as online adaptive radiotherapy.

Previous studies have laid foundations for online ART training, with Shepherd et al. [12] providing a framework for Radiation Therapist (RT) expertise and credentialing and Shepherd et al. [13] proposing a global, multidisciplinary curriculum. Our study extends this foundation by incorporating incident-informed examples within simulation platforms to train and credential the entire ART team.

The aim of this work was to develop and implement a multi-institutional, incident-informed training program for online adaptive radiotherapy (ART) teams that provides practical experience in error recognition, troubleshooting, and mitigation within a simulated clinical setting. Using retrospective patient treatment data, we recreated real incidents in an interactive emulator-based platform. This approach aims to improve quality assurance skills of the ART team, reduce workflow errors, and enhance patient safety and treatment quality in online ART.

Furthermore, to promote knowledge exchange and create a realistic learning environment, we combined expertise from a center with extensive ART experience [14] and a center in the early stages of implementing an ART program.

The novelty of this work lies not in the emulator technology itself, but in the systematic recreation of real-world ART incidents and integration of team training into a multi-institutional framework. To our knowledge, this is the first incident-driven simulation approach for training of online ART teams.

## Methods

Three cases are presented in this retrospective study, selected from 41 incident reports involving patients treated with CT-guided ART on the Ethos system (Varian Medical Systems, Palo Alto, CA) under an approved protocol by Ethics Committee/ Institutional Review Board (IRB). Informed consent was waived due to the retrospective quality assurance nature of the study. These incidents were collected in 2024 from the institutional process-improvement reporting system and from experiences shared by adaptive team members. The process-improvement records provided a detailed account of each incident, including the nature of the event, contributing factors, the stage of the workflow at which it occurred, and whether the incident reached the patient.

Across the two participating institutions, eight adaptive team members reviewed all 41 incidents, categorized them by underlying cause (patient-related, user-related, or system-related), and retrospectively scored each incident. The team was comprised of seven medical physicists with varying experience levels (2 early-career, 2 mid-career, and 4 with extensive adaptive radiotherapy experience) and one experienced advanced radiation therapist. Incidents were graded for the severity of their dosimetric impact on the treatment plan, as well as for frequency of occurrence and detectability within the team, to calculate the Risk Priority Number (RPN). Because of varying levels of expertise and clinical exposure among team members, the retrospective RPN scoring primarily served as a training exercise to enhance understanding of perceived risk within a retrospective FMEA framework, rather than representing a formal prospective FMEA implementation.

Table 1 provides a summary of all recorded incidents. Severity was scored on a 1–10 scale based on TG-100 FMEA principles and reflects the potential clinical impact if a failure remains undetected (1–3: minimal impact; 4–6: moderate deviation; 7–8: major risk to target coverage or Organ at Risk (OAR) constraints; 9–10: critical potential for serious patient harm). The final RPN for each incident was calculated as the average of the scores assigned by all team members. Three incidents (shown in bold in Table 1), representing a range of high RPN, high severity, and high number of reported incidents (with average RPN and severity), were selected for our emulator-based simulation.

**Table 1:** Summary of identified ART-related incidents. The “# of Incidents” column reflects the number of reported similar incidents among the 41 reviewed cases.

**# of Incidents**	Abstract Description.	**Related Category**	**RPN**	**Severity**
**Five**	**Wrong arm in treatment setup**	**User**	**62**	**4**
One	Hole in BODY contour away from target	System	12	3
One	Fiducials not contoured	User	7	3
Two	Confusing structure naming post-Whipple	User	14	3
One	Poor auto-contouring	System	34	3
Three	Photon starvation in iCBCT scan	User	49	4
One	Plan revision due to target growth; ContourRing outdated	User	99	6
One	Scheduled plan not created	System	6	2
Three	OAR objective unmet in first adapted plan run; re-optimization	System	29	5
Three	OAR under-contoured within ContourRing	User	120	7
**Six**	**OARs moved into PTV_Opt on pre- or mid-treatment scans**	**Patient**	**172**	**6**
One	Patient abdominal muscle contraction affecting breath hold	Patient	109	4
One	Inactive derived structures	User	74	7
Four	Patient setup off by > 5 cm from treatment isocenter	User	94	5
One	Prostate misaligned with fiducials	User	55	6
One	Rectal gel near GTV	User	107	7
Two	Inconsistent patient breathing causing multiple CBCT corrections	Patient	53	6
One	Setup appeared correct, but large gas present in OARs	Patient	34	4
**One**	**Target drawn outside ContourRing**	**User**	**96**	**9**
One	Very high MU difference; re-optimization by covering MD	System	24	5
One	Two targets with one isocenter; one target moved mid-treatment	Patient	41	6

Although the reported incidents involved multiple treatment sites, the majority occurred in pancreatic cases. Given the anatomical and workflow complexity of pancreas treatments in the online adaptive setting, pancreas cases were selected as representative scenarios to illustrate the incident categories and their potential dosimetric consequences.

Representative data were anonymized, and the planning CTs, RT structures, and the treatment-day cone beam computed tomography (CBCTs) were imported into the Ethos emulator. Clinical constraints and planning objectives from the original treatment plans were also transferred to the emulator as a template to ensure consistency with standard treatment practices. A simulated online adaptive workflow was then performed, including contouring on the anatomy of the day, plan re-optimization, and adaptive plan review prior to virtual treatment selection. Plans were generated using the Ethos treatment planning system (TPS) with the Acuros XB dose calculation algorithm (Varian Medical Systems, Palo Alto, CA) and a 2 mm calculation grid size. Dosimetric impacts were evaluated using target coverage, OAR dose metrics, and dose–volume histograms (DVHs).

All three scenarios presented here represent stereotactic body radiotherapy (SBRT) plans for pancreatic tumor, with a prescription dose of 50  Gy in 5 fractions. Institutional OAR constraints for the stomach, duodenum, small bowel, and large bowel were applied, with D0.5  cc < 33  Gy. Patients were positioned supine and immobilized using a Vac-Lok device.

### Case 1: incorrect target delineation (User-related)

During one online ART session, a portion of the gross target volume (GTV) was inadvertently contoured outside the designated contour ring. This target delineation error was not detected before treatment delivery and was identified on the mid-treatment verification scan during contour review.

To simulate this scenario, a representative pancreatic treatment plan was recreated in the Ethos emulator. An anonymized CBCT dataset was used with the patient in the treatment position (left arm up and right arm by the side). During the emulator session, the target contour was deliberately modified on several slices to extend beyond the contour ring. Online adaptive planning was then performed, and the resulting plan was evaluated for the dosimetric impact on target coverage and surrounding organs at risk (OARs), including the liver (overlapping the incorrectly contoured target), stomach, large bowel, small bowel, and duodenum.

### Case 2: intra-fractional OAR motion into high dose region (Patient-related)

On the treatment day, a planning CBCT scan was acquired and the target and OARs were delineated to generate an online adaptive plan. After the ART team reviewed and approved the adapted plan, a pre-treatment verification CBCT was obtained to confirm that patient alignment and anatomy matched the planning scan. Between the planning and verification CBCTs, the stomach had shifted into the PTV_OPT, where higher dose is allowed because this region does not overlap with OARs. Following the institutional ART practice, the patient was re-scanned after 5 minutes to allow for potential spontaneous OAR repositioning; however, the repeat scan showed no change in stomach location. Due to the risk of OAR overdose, the ART team elected to abort treatment for that fraction.

To simulate this scenario, the representative pancreatic SBRT case described above was recreated in the Ethos emulator. Planning and verification CBCTs were contoured separately to reflect the observed variations in OAR position and deformation. The dose from the planning CBCT was recalculated on the verification CBCT without re-optimization to evaluate the potential dosimetric impact of unaccounted anatomical changes. The resulting plan was used to assess effects on OAR dose metrics (stomach and small bowel) and PTV coverage.

### Case 3: wrong arm position (User-related)

During simulation CT acquisition for a pancreatic case, the patient was initially positioned with the left arm down and right arm up. Due to the tumor laterality, the treatment plan was designed using left-sided beams, and the arm position was intentionally changed for treatment (left arm up, right arm down) to avoid beam interference and minimize dose to adjacent OARs. To accommodate the intended arm-up position in the TPS, the left arm was excluded from the body contour during planning.

The arm-position change was documented in the adaptive physics worksheet during offline plan review, and the plan report included an axial image demonstrating the left-sided beam arrangement. However, on the treatment day, this positional change was overlooked and the patient remained with the left arm down.

To simulate this scenario, an anonymized CBCT with the left arm down was used in the Ethos emulator. A simulated online adaptive workflow was performed, generating two adaptive plans on the same CBCT dataset to model the dosimetric impact of the intended arm-up versus the incorrect arm-down position. For the arm-up scenario, the body contour was modified to exclude the left arm, consistent with the planned beam geometry. Dose metrics were then compared between the plans to evaluate the impact of arm position on target coverage and surrounding OARs, including the stomach, bowel, and kidney.

## Results

**Fig. 1:**
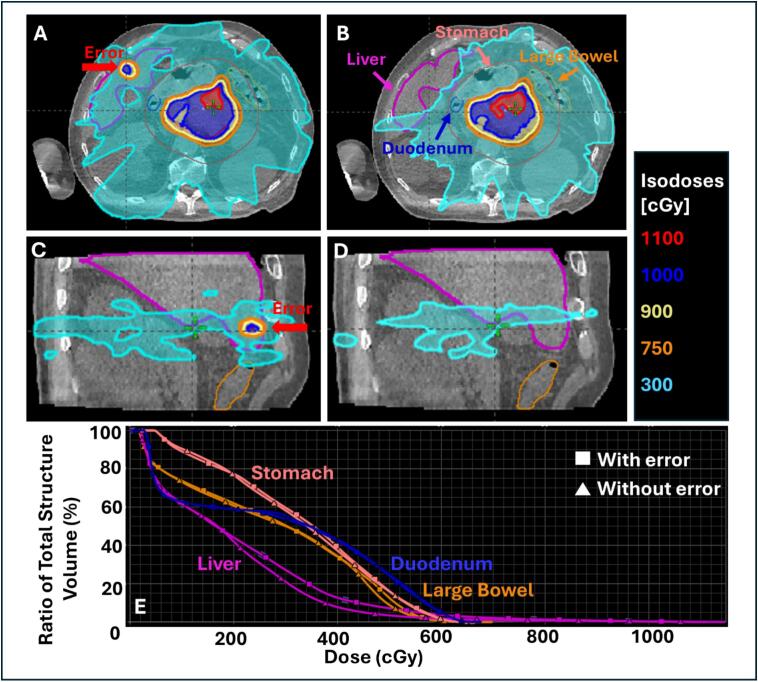
Panels **A** and **C** show axial and sagittal dose maps, respectively, demonstrating the dosimetric effect of the target delineation error. Panels **B** and **D** show the corresponding dose distributions without the target delineation error. Panel **E** presents dose–volume histogram (DVH) comparisons for the stomach, duodenum, large bowel, and liver for plans with and without the contouring error.

For Case 1, the target delineation error resulted in a portion of the liver outside the contour ring receiving the full prescribed per-fraction target dose (1000  cGy). Fig. 1 illustrates the dosimetric impact per fraction for a total prescribed dose of 5000  cGy delivered in five fractions.

The clinical significance of such an incident depends on (1) the radiosensitivity of the affected organ and (2) the magnitude of the dose received. OAR overdose under these circumstances could increase the risk of treatment-related toxicity. In practice, the delivered dose to OARs is evaluated by the treating physician, who determines whether planning modifications are necessary for subsequent fractions. In this case, because the liver was not in close proximity to the target, no additional actions were required to reduce liver dose in the subsequent fractions.

Although the incident was identified mid-treatment, for training purposes, the dosimetric consequences are illustrated assuming the full fraction dose had been delivered.

**Fig. 2:**
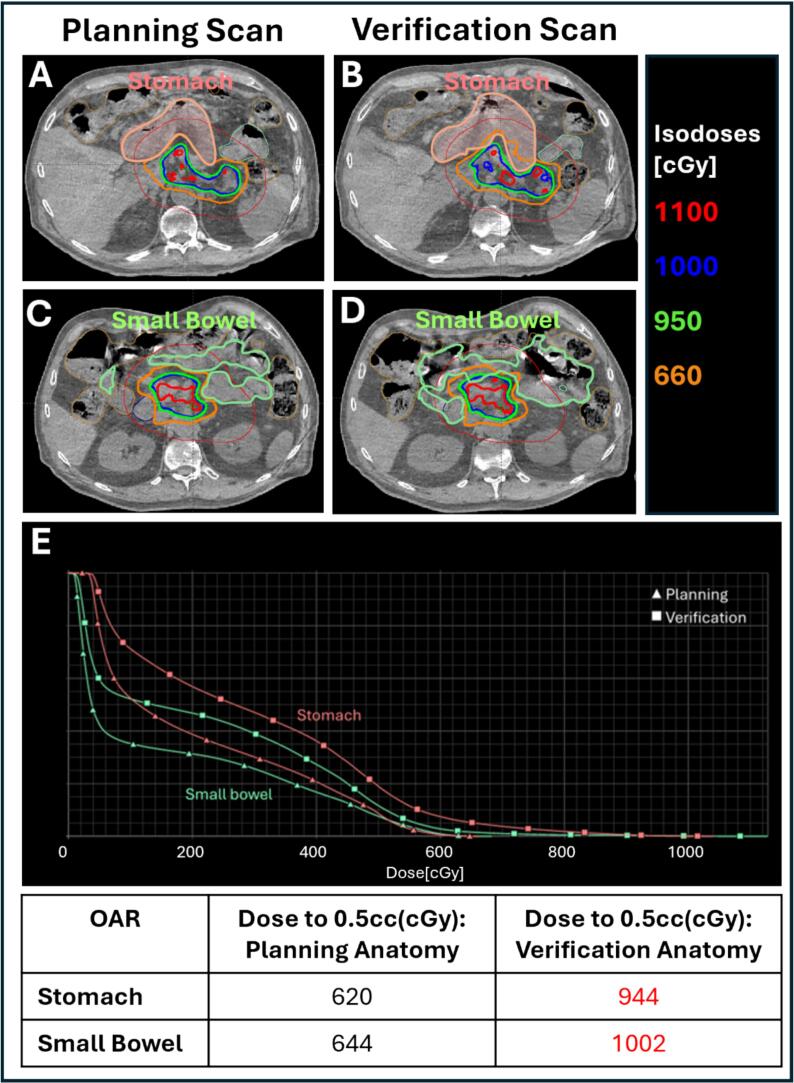
Dose distributions from the planning and verification CBCTs illustrating OAR dose exceedances due to intra-fraction OAR repositioning observed on the verification CBCT. Panels A–B show stomach dose, and panels C–D show small bowel dose, with both exceeding tolerance levels. Panel E presents DVH curves for the stomach and small bowel from the planning and verification scans. The corresponding dose metrics for both organs are summarized in the bottom panel, with values exceeding OAR tolerance highlighted in red. (For interpretation of the references to colour in this figure legend, the reader is referred to the web version of this article.)

For Case 2, failure to re-optimized the plan for anatomical changes between the planning and pre-treatment verification scans, resulted in an overall increase in OARs dose (Fig. 2). The small bowel received 1002 cGy (342  cGy above the per-fraction constraint of 660  cGy), exceeding tolerance by more than 50%, while the stomach received 944 cGy (284  cGy above the same constraint), surpassing tolerance by over 40%. These results illustrate that delivering treatment without adapting to anatomical changes could substantially increase the risk of OAR toxicity. In the actual clinical scenario, however, the treatment fraction was aborted after the verification CBCT identified the significant anatomical change, and no dose was delivered to the patient.

**Fig. 3:**
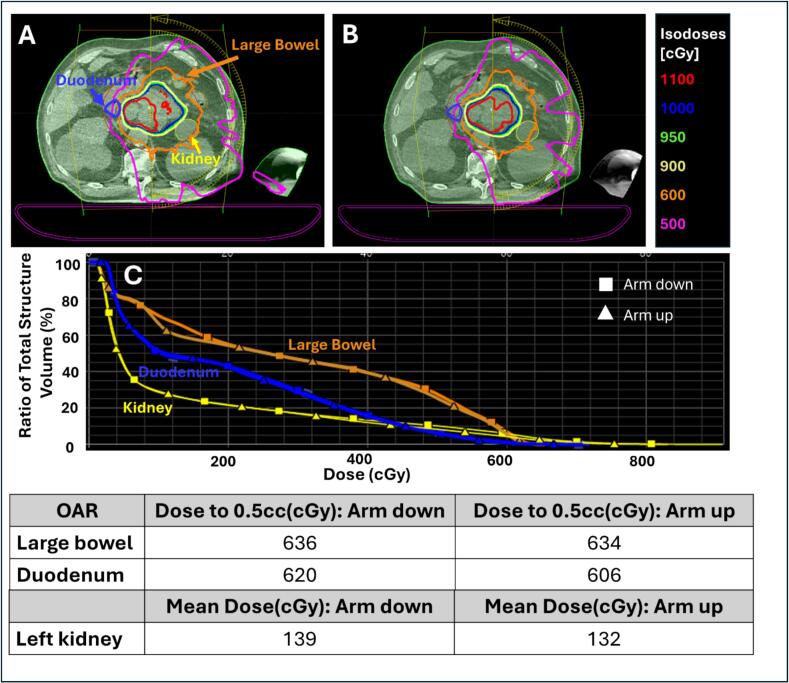
Dose distributions illustrating the effect of wrong arm positioning (arm down) on OAR dose. Panel **A** shows the dose map overlaid on patient anatomy with the arm-down position, while panel **B** shows the corresponding dose distribution with the arm-up position. Panel **C** presents DVH comparisons for the large bowel, duodenum, and left kidney for the arm-up versus arm-down positions. The bottom panel summarizes the tabulated planning metrics for OARs affected by the arm-down position.

In Case 3, improper left arm positioining resulted in increased OAR doses along the left-sided beam paths. Specifically, D0.5 cc to the large bowel and duodenum increased by 2 cGy and 14 cGy per fraction, respectively, while mean left kidney dose increased by 7 cGy. The arm also received unnecessary dose. As illustrated in Fig. 3, proper arm positioning is therefore critical for maintaining the intended dose distribution and adhering to ALARA principles. Although the change in arm position resulted in increased OAR dose, the magnitude was not considered clinically significant. Following physician review, no modifications to the treatment plan or subsequent fractions were required.

## Discussion

This study provides a foundation for incident-informed ART team training by recreating representative failure scenarios in an emulator-based platform and evaluating their dosimetric impact. The resulting case-based materials allow team members to visualize how errors affect dose distributions and practice mitigation strategies in a controlled environment. The lessons learned from the case scenarios are summarized below.

**Case 1 – Target Delineation Error:** This scenario highlights the importance of carefully reviewing all contours to ensure the target is accurately defined and confined within the contour ring. During online plan evaluation, the team should verify that dose falloff outside the contour ring is appropriate, including reviewing low-dose isodose lines (e.g., 10% Rx) to detect any unintended high-dose regions. An additional safeguard is to create a BODY-minus-contour-ring structure with a high-priority maximum-dose constraint, which should remain below 50% of the prescription dose. These steps help prevent unintended dose to adjacent organs. This scenario can also be used in emulator-based training to allow staff practice detecting contouring errors and applying corrective actions, reinforcing skills alongside procedural safeguards.

**Case 2 – OAR Migration into PTV_Opt:** Disregarding OARs that shift into the PTV_Opt can result in significant unintended dose. If minimal patient shifts cannot displace the OAR while maintaining target coverage, the team should wait briefly and acquire a repeat scan to assess spontaneous repositioning. If slight overlap persists, the physician may decide to treat for that single fraction, acknowledging a minor OAR overdose, and adjust contouring in subsequent fractions to balance cumulative dose. Given the high mobility of small and large bowel relative to more stable organs such as the stomach and duodenum, intra-fraction dose predictions may be less reliable. When substantial anatomical changes occur, the safest approach is to restart the adaptive session and re-optimize the plan.

**Case 3 – Arm-Positioning Error:** Proper patient setup is critical for maintaining intended dose distributions. The ART team should verify that arm positioning matches the planned beam geometry and laterality, at both simulation and during pre-treatment imaging. Reviewing target location during simulation can guide proper arm positioning and reduce the risk of beam interference with OARs and unintended OAR dose. Even small setup deviations, as demonstrated in this case, can increase dose to structures along the beam path, highlighting the importance of consistent, documented positioning.

**Clinical Significance of Incident Dose Deviation:** Although the dosimetric deviations described in these cases occurred within a single fraction, their potential clinical significance depends on both the magnitude of the dose deviation and the radiosensitivity of the affected organ. In hypofractionated treatments such as pancreatic SBRT, even a single fraction exceeding OAR constraints may increase the risk of toxicity, particularly for serial organs such as the stomach or duodenum. However, the greater clinical concern arises if similar errors remain undetected and are repeated across multiple fractions, potentially leading to cumulative dose violations and increased toxicity risk. These scenarios highlight the importance of early detection within the adaptive workflow, where verification imaging, contour review, and team-based plan evaluation serve as safeguards to prevent systematic propagation of errors throughout the treatment course.

This study was not designed to serve as a comprehensive audit of institutional incident management or regulatory reporting. When applicable, patient notification and institutional follow-up were carried out according to established clinical and regulatory policies. The reported dose deviations are presented per fraction to illustrate potential dosimetric impact; specific OAR dose thresholds may vary across institutions and regions. The primary aim of this work was to develop a simulation-based, incident-informed training platform that enables ART teams to visualize dosimetric impact and practice mitigation strategies in a controlled educational setting, rather than to evaluate compliance with institutional or national dose constraints.

**Institutional Variations and Scoring**: As shown in Table 1, differences in the frequency of incident occurrence across institutions resulted in variations in RPN values. For example, incidents involving incorrect arm positioning during treatment occurred more frequently at one institution than another. This discrepancy arose because one institution’s policy was to maintain the same arm position from simulation to treatment (both arms up), rather than switching the arms. These findings suggest that ART incidents are largely institution specific. Therefore, each institution should implement ART quality assurance guidelines tailored to their own institutional FMEA.

**Study Achievements, Limitations, and Future Directions**: This work represents an initial step toward a systematic, incident-informed framework for ART team training. By reviewing reported incidents, recreating representative failure scenarios, and evaluating dosimetric impacts, we developed case-based learning modules that actively engage ART teams in practicing error recognition and mitigation. Preliminary implementation at participating institutions has included emulator-based hands-on training, internal presentations, adaptive workflow triage meetings, and periodic refresher sessions to reinforce awareness of potential failure points within the online adaptive workflow.

**Role of Emulator-Based Simulation in ART Training:** Compared with traditional training approaches such as lectures, written protocols, or case reviews, emulator-based simulation provides a near-realistic environment in which the full online adaptive workflow can be reproduced. Team members can perform contour review, plan re-optimization, and plan evaluation using the same interface and workflow used in clinical practice. This hands-on experience allows staff to practice recognizing potential errors and applying mitigation strategies in situations that closely resemble real clinical incidents. As a result, the training can promote deeper understanding of the adaptive workflow and strengthen team preparedness for managing unexpected scenarios during online ART.

The educational activities are currently implemented on a limited scale, lack standardization across institutions, and have not yet been formally evaluated for their impact on team performance or patient safety. Future work will focus on systematically assessing how these training activities influence ART team competence, workflow adherence, and patient outcomes, as well as developing standardized protocols to optimize the effectiveness of incident-informed training. The next phase will also explore structured integration of these materials, including routine triage of missed or challenging cases, selection of individual team members for hands-on scenario practice, and the creation of measurable endpoints to track improvement in team performance and mitigation of potential errors.

While this study focused on the CT-guided Ethos platform, the methodology of incident-informed team training can be extended to other adaptive and non-adaptive radiotherapy workflows. MR-guided online ART, in particular, could greatly benefit from this approach due to its increased workflow complexity, reduced automation, and more reliance on user-dependent quality assurance checks, which introduce additional opportunities for errors.

## Conclusion

This study presents the development of an incident-informed, simulation-based training framework for adaptive radiotherapy (ART) teams. By recreating real-world ART incidents in an emulator environment, we generated case-based training scenarios that illustrate the dosimetric impact of workflow errors and highlight practical mitigation strategies. These materials provide an educational platform that allows ART team members to practice recognizing and troubleshooting potential failures within the online adaptive workflow. Beyond individual training, this incident-informed approach can also stimulate multidisciplinary discussion on institutional policies, system design, and workflow improvements. Future work will focus on broader implementation and formal evaluation of this training framework to assess its impact on team performance, workflow adherence, and patient safety in online ART.

## Declaration of competing interest

The authors declare the following financial interests/personal relationships which may be considered as potential competing interests: **Geoffrey Hugo, PhD** has received **research funding** paid to his institution from Varian Medical Systems, Siemens Healthineers, and Radformation and **consulting fees** from Varian Medical Systems. He also holds **patents**, planned, issued, or pending licensed to Varian Medical Systems. All the other authors declare no conflicts of interest related to this work.
